# Diagnostic significance of adenosine deaminase in pleural effusate for primary effusion lymphoma–like lymphoma

**DOI:** 10.1002/ccr3.3296

**Published:** 2020-10-06

**Authors:** Soichiro Nakako, Mitsushige Nishimura, Yoshiro Murakami, Atsuko Mugitani

**Affiliations:** ^1^ Department of Hematological Medicine Osaka City University Hospital Osaka Japan; ^2^ Department of General Internal Medicine Fuchu Hospital Izumi Japan; ^3^ Department of Hematological Medicine Fuchu Hospital Izumi Japan

**Keywords:** adenosine deaminase in pleural effusion, primary effusion lymphoma–like lymphoma, tuberculous pleural effusion

## Abstract

Our case and review of the literature suggests the possibility that pleural effusate adenosine deaminase levels in patients with primary effusion lymphoma–like lymphoma (PEL‐LL) might be much higher than those in patients with other types of lymphomas and those with tuberculous pleural effusion.

## Graphical abstract

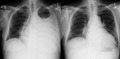

## 1 INTRODUCTION

We experienced a case of primary effusion lymphoma–like lymphoma with very high pleural effusate adenosine deaminase (ADA) levels. We performed a literature search to test our hypothesis that pleural effusate ADA levels might be very high in these patients as compared to other lymphoma and tuberculous pleural effusion patients.

Tuberculous pleural effusion and lymphoma are included in the differential diagnosis of patients with high adenosine deaminase (ADA) levels in pleural effusate. Previous reports showed that median ADA levels in pleural fluid were 73 IU/L (interquartile range 44‐124 IU/L) and 84 IU/L (interquartile range 64‐110 IU/L) in patients with lymphoma‐associated malignant effusion (n = 17) and tuberculous pleural effusion (n = 216), respectively.^1^ Our patient with primary effusion lymphoma (PEL)–like lymphoma (PEL‐LL) had extremely high ADA levels in pleural fluid. We hypothesized that pleural effusate ADA levels in patients with PEL‐LL might be much higher than in those with other types of lymphoma and tuberculous pleural effusion. We compared our case with previous cases of PEL‐LL obtained using a PubMed search.

## 2 CASE

A 74‐year‐old man presented to the cardiology department of our hospital because of dyspnea on exertion for 4 days and weight gain of 5 kg over week. His past medical history included chronic heart failure due to cardiac amyloidosis, hypertension, deep venous thrombosis, chronic kidney disease, and dyslipidemia. He did not have any relevant family history. He was under treatment with the vasopressin receptor antagonist tolvaptan 7.5 mg, oral anticoagulant edoxaban 30 mg, bezafibrate 200 mg to treat hyperlipidemia, and the beta‐blocker bisoprolol 2.5 mg.

**Figure 1:**
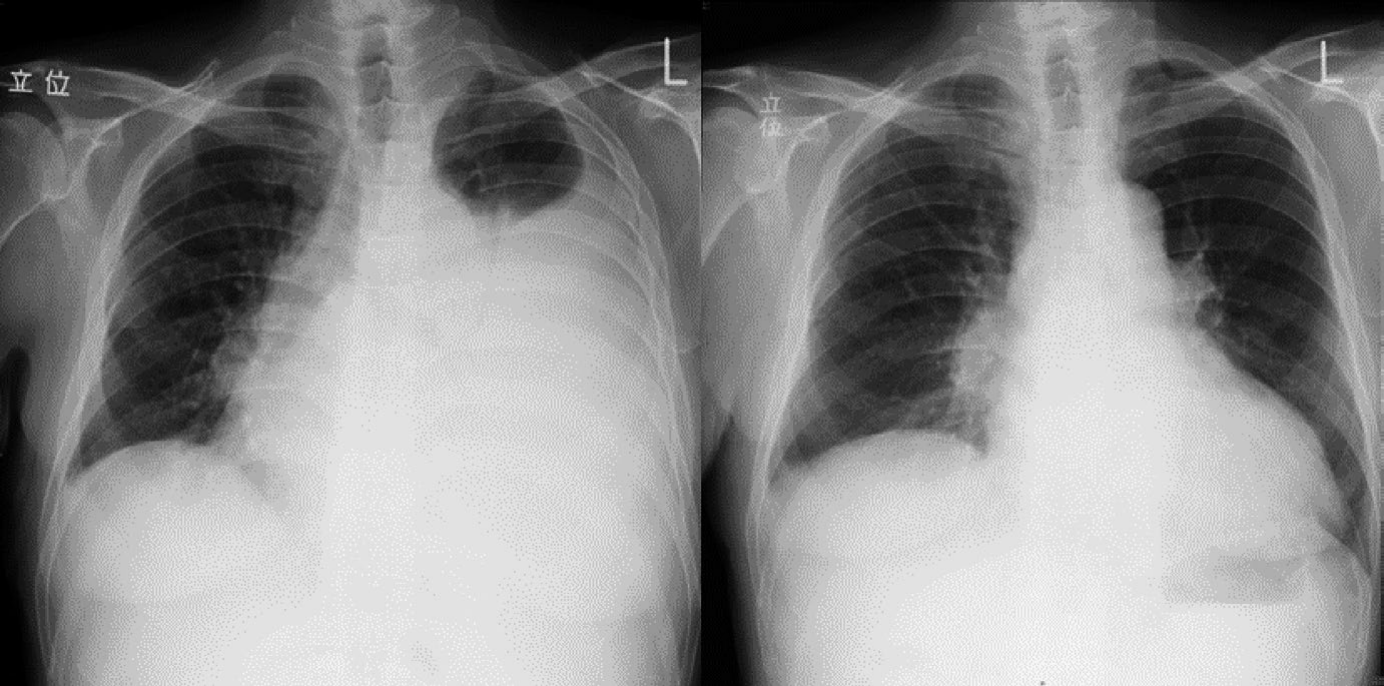
Left: Chest X‐ray on admission. Right: Chest X‐ray after treatment

Upon admission to the hospital, his dyspnea worsened and was present at rest. Chest X‐ray showed left pleural effusion and mediastinal shift to the right (Figure 1). Chest computed tomography (CT) showed left pleural effusion and no lymphadenopathy. Echocardiography showed no pericardial effusion and normal cardiac function. Thoracentesis was performed and a total of 3300 mL of hemorrhagic pleural fluid was drained over a period of 4 days. Dyspnea improved after the drainage, and he was discharged 1 week later. Pleural fluid analysis showed increased number of atypical lymphocytes and the effusion recurred gradually after discharge, so he was referred to our hematology department one month after he left the hospital.

On presentation to our department, he had dyspnea on exertion and weight gain. He had slight fever, night sweats, loss of appetite, malaise, and chest pain. His vital signs indicated a body temperature of 36.5°C, blood pressure of 134/80 mm Hg, pulse rate of 87 beats/min, and respiratory rate of 18 breaths/min, and his oxygen saturation was 99% on ambient air. He had no conjunctival pallor. Jugular venous distension was not observed. Breath sounds were diminished on the left side, and heart sounds were normal. He had edema in both lower extremities.

### 2.1 Differential diagnosis, investigation, and treatment

Laboratory findings showed a white blood cell count of 3.5 × 10^9^/L, hemoglobin of 109 g/L, platelet count of 128 × 10^9^/L, aspartate aminotransferase of 32 IU/L (normal range: 13‐31 IU/L), alanine aminotransferase of 13 IU/L (normal: 8‐34 IU/L), lactate dehydrogenase (LDH) of 209 IU/L (normal: 115‐217 IU/L), serum protein of 6.2 g/dL (normal: 6.6‐8.1 g/dL), serum albumin of 3.6 g/dL (normal: 4.1‐5.1 g/dL), C‐reactive protein of 0.11 mg/dL (normal: 0.00‐0.14 mg/dL), serum creatinine of 1.19 mg/dL (normal: 0.65‐1.07 mg/dL), blood urea nitrogen of 12.6 mg/dL (normal: 8‐20 mg/dL), and sodium of 141 mEq/L (normal: 136‐147 mEq/L). Interferon γ‐release assay for *Mycobacterium tuberculosis*, and tests for hepatitis B surface (HBs) antigen, HBs antibody, hepatitis B core antibody, hepatitis C virus antibody, human immunodeficiency virus (HIV) antigen, HIV antibody, and human T‐cell lymphotropic virus type 1 antibody were all negative. Pleural fluid analysis showed a pH of 7.6, cell count of 26,932/μL (segmented cells 2.0%, lymphocytes 7.5%, atypical lymphocytes 90.5%), protein of 4.1 g/dL, albumin of 2.5 g/dL, glucose of 13 mg/dL, LDH of 683 IU/L, and ADA level of 140.7 IU/L. Pleural fluid polymerase chain reaction (PCR) testing for tuberculosis was negative. Pleural fluid culture did not grow any pathogens. Cytology showed atypical lymphocytes. Pleural fluid samples were positive for immunoglobulin gene rearrangement. Flow cytometry was positive for CD19, CD20, and CD79a and was negative for CD3, CD10, and CD23. Pleural fluid was negative for human herpesvirus‐8 (HHV‐8) DNA. Whole‐body CT showed no malignant tumor, ascites, or lymphadenopathy.

In this patient, complete blood count and metabolic panel did not show any specific findings. Chest X‐ray showed evidence of massive pleural effusion, but whole‐body CT showed no malignant tumor, lymphadenopathy, or ascites. Pleural fluid analysis showed elevated atypical lymphocytes, ADA, and LDH.

Based on these results, our differential diagnoses included heart failure due to amyloidosis, tuberculous pleural effusion, and pleural effusion related to malignant lymphoma. His cardiac function was normal on echocardiogram and there were no symptoms suggestive of heart failure, as indicated by the Framingham heart failure diagnostic criteria. Tuberculous pleural effusion was ruled out because pleural fluid acid‐fast bacillus smear, culture, and PCR for tuberculosis were negative. Whole‐body CT scan showed no lymphadenopathy. Hence, we narrowed down the diagnosis to PEL and PEL‐LL. The positive immunoglobulin gene rearrangement and flow cytometry results mentioned above suggested B‐cell lymphoma. Moreover, our patient did not have lymphadenopathy or HHV‐8 DNA in the pleural fluid. Based on these findings, we made a final diagnosis of PEL‐LL.

Some of the treatment options for PEL‐LL have been reported in previous case reports. However, there have been no comparative studies describing definitive treatment. We decided to give weekly rituximab, which is reported to have low toxicity in elderly patients.^2^ We performed thoracentesis for drainage of the pleural effusate and administered 4 cycles of weekly rituximab (375 mg/m^2^).

### 2.2 Outcome and follow‐up

The efficacy of treatment was assessed based on the patient's symptoms, physical examination, body weight, and chest X‐ray. He underwent one thoracentesis procedure for drainage and 4 cycles of weekly rituximab. He received no further treatment after the drainage and chemotherapy. He has been visiting our hospital every 2 weeks since his discharge. During this period, he has not complained of any more symptoms and chest X‐ray has not shown a recurrence of pleural effusion (Figure 1). He has been in complete remission for 6 months after discharge.

## 3 DISCUSSION

PEL is defined by the World Health Organization classification system as a type of malignant lymphoma in which tumor cells are only found in body cavity fluids, without lymphadenopathy, and is associated with HHV‐8 infection. PEL‐LL has the same characteristics as PEL and is diagnosed based on evidence of immunoglobulin gene rearrangement and flow cytometry, and the absence of HHV‐8 infection.

We collected previous cases of PEL‐LL using a PubMed search to assess our hypothesis. A PubMed search using the keyword “Primary effusion lymphoma like lymphoma” filtered by the words “Case report”, “Full text”, and “English” identified 47 cases. However, only 7 of the 47 cases that we reviewed described pleural fluid ADA levels. We have presented data on the ADA levels of a total of 9 cases in Table 1, including these 7 cases and our present and previous case.^2^, ^3^, ^4^, ^5^, ^6^, ^7^

**Table 1:** Adenosine deaminase (ADA) levels of our cases and those in previous literature

Section References	Age	Sex	ADA in pleural effusion(IU/L)
2	90	Male	101.3
2	87	Female	477
3	80	Female	130
4	86	Male	110.5
5	77	Male	310
6	86	Male	113
7	73	Female	316.8
Our previous case	87	Male	102.9
Our present case	74	Male	140.7

Abbreviation: ADA, adenosine deaminase.

The lowest and highest ADA levels were 101.3 IU/L and 477 IU/L, respectively, and the mean value was 225.3 IU/L. This level was much higher than ADA levels previously reported in lymphoma cases with pleural effusion and tuberculous pleural effusion cases.^1^ Our case and review of the literature suggests the possibility that pleural effusate ADA levels in patients with PEL‐LL might be much higher than those in patients with other types of lymphomas and those with tuberculous pleural effusion. More data are needed to validate our observation. In the future, our report might serve as a cornerstone for determining whether or not pleural effusion ADA levels vary among lymphoma types.

## CONFLICT OF INTEREST

None declared.

## AUTHOR CONTRIBUTIONS

SN: wrote the manuscript. MN: collected and analyzed the data. YM: collected and analyzed the data. AM: supervised the study.

## INFORMED CONSENT

We confirmed patient consent by written format which was added to submitted documents.
